# Identification of proteomic markers for prediction of the response to 5-Fluorouracil based neoadjuvant chemoradiotherapy in locally advanced rectal cancer patients

**DOI:** 10.1186/s12935-022-02530-0

**Published:** 2022-03-15

**Authors:** Jianan Wang, Jiayu Liu, Jinyang Wang, Shijian Wang, Feifei Li, Ruibing Li, Peng Liu, Mianyang Li, Chengbin Wang

**Affiliations:** 1 Department of Laboratory Medicine, The First Medical Centre of Chinese PLA General Hospital, Beijing, 100853 China; 2 Medical School of Chinese PLA, Beijing, 100853 China; 3 School of Laboratory Medicine, Weifang Medical College, Weifang, 261053 Shandong China; 4 Nankai University School of Medicine, Nankai University, Tianjin, 300071 China; 5 Department of Pathology, The First Medical Centre of Chinese PLA General Hospital, Beijing, 100853 China

**Keywords:** Locally advanced rectal cancer, Neoadjuvant chemoradiotherapy, Predictive marker, Proteomics

## Abstract

**Background:**

Neoadjuvant chemoradiotherapy (nCRT) prior to surgery is the standard treatment for patients with locally advanced rectal cancer (LARC), while parts of them show poor therapeutic response accompanied by therapy adverse effects. Predictive biomarkers for nCRT response could facilitate the guidance on treatment decisions but are still insufficient until now, which limits the clinical applications of nCRT in LARC patients.

**Methods:**

In our study, 37 formalin-fixed paraffin-embedded tumor biopsies were obtained from patients with LARC before receiving 5-fluorouracil based nCRT. Proteomics analyses were conducted to identify the differentially expressed proteins (DEPs) between total responders (TR) and poor responders (PR). The DEPs were validated via ROC plotter web tool and their predictive performance was evaluated by receiver operating characteristic analysis. Functional enrichment analyses were performed to further explore the potential mechanisms underlying nCRT response.

**Results:**

Among 3,998 total proteins, 91 DEPs between TR and PR were screened out. HSPA4, NIPSNAP1, and SPTB all with areas under the curve (AUC) ~ 0.8 in the internal discovery cohort were independently validated by the external mRNA datasets (AUC ~ 0.7), and their protein levels were linearly correlated with the graded responses to nCRT in the internal cohort. The combination of HSPA4 and SPTB could distinctly discriminate the TR and PR groups (AUC = 0.980, *p* < 0.0001). Moreover, multiple combinations of the three proteins realized increased specificity and/or sensitivity, while achieving favorable predictive value when moderate responders were introduced into the ROC analysis. Pathways including DNA damage repair, cell cycle, and epithelial mesenchymal transition were involved in nCRT response according to the enrichment analysis results.

**Conclusions:**

HSPA4, SPTB and NIPSNAP1 in tumor biopsies and/or their optional combinations might be potential predictive markers for nCRT response in patients with LARC. The DEPs and their related functions have implications for the potential mechanisms of treatment response to nCRT in patients with LARC.

**Supplementary Information:**

The online version contains supplementary material available at 10.1186/s12935-022-02530-0.

## Background

Colorectal cancer (CRC) ranked third in terms of incidence, but second in terms of mortality among all cancer types. Accounting for 30–35% of CRC cases, rectal cancer (RC) was estimated to cause over 0.73 million new cases and ~ 0.34 million deaths in 2020 [1]. Due to the late detection or delayed diagnosis, patients are often diagnosed with locally advanced rectal cancer (LARC) in which the tumor has grown into the outermost layers (AJCC T3) or through the rectum wall into local adjacent structures (AJCC T4), with or without positive regional lymph node metastases.

LARC is regularly treated with trimodality therapy comprising preoperative neoadjuvant chemoradiation therapy (nCRT), surgery (total mesorectal excision; TME), and adjuvant chemotherapy. The standardized regimen of nCRT includes neoadjuvant radiotherapy and concomitant chemotherapy with the intravenous 5-fluorouracil (5-FU) or its oral analogue such as capecitabine. Benefits of this approach include significant tumor downsizing, downstaging, and the potential for achieving pathologic complete response (pCR), therefore improving resectability, anal sphincter preservation, local control and survival after radical surgery in patients with LARC [2–5]. Despite the effective trimodality therapy, the response to nCRT varies among patients with LARC, which is associated with long-term outcomes including recurrence-free survival, distant metastasis, and local recurrence rates [4]. Complete response is found in only about 15 ~ 18% of the LARC patients [4–7], while a subset of patients (~ 20%), being treated with the same regimen, do not achieve a favorable response —suggesting potential resistance to nCRT. LARC patients who are evaluated to be resistant to nCRT would be supposed to receive optimized treatment earlier in their clinical management. Therefore, there is a strong need for predictive biomarkers to identify the subsets of patients who are resistant or sensitive to nCRT before therapy, which would certainly benefit personalized treatment strategies for patients with LARC.

Clinical factors including tumor size, T and N stage, pathological features and imaging modalities have been shown to facilitate the prediction of the response to nCRT; however, their clinical application is limited due to the moderate sensitivity and specificity in prediction [8–13]. Based on the literature, tissue-derived molecular biomarkers involved in DNA mutation and methylation, gene expression profiles, protein and metabolites, the tumor immune microenvironment, and microRNAs have been considered for their potential to predict nCRT response early with promising efficacy [8]. Nevertheless, few viable biomarkers have reached clinical application.

At present, the standard assessment for rectal cancer response to neoadjuvant therapy is the tumor regression grade (TRG). Among existing TRG systems, the American Joint Committee on Cancer (AJCC) TRG system has shown superior prediction for survival outcomes [14]. In this study, we grouped patients with LARC into different response groups according to the AJCC TRG system. Then, proteins were extracted from formalin-fixed paraffin-embedded (FFPE) biopsies of patients with LARC before nCRT, before analyzing with liquid chromatography with tandem mass spectrometry (LC–MS/MS) to characterize the tumor proteomic signature and identify the differentially expressed proteins (DEPs). The signaling pathways these DEPs involved in were explored to gain an insight into their potential roles in treatment response. Furthermore, external mRNA datasets were used to verify the levels of DEPs in different response groups, and the predictive abilities of verified DEPs were assessed using receiver operating characteristic (ROC) curves, laying the groundwork for future clinical application.

## Methods

### Patients and samples

Our study finally enrolled 42 patients diagnosed with LARC (clinical stage T3–4N0 or T1–4N1–2) and treated with pre-operative 5-FU-based nCRT followed with surgery from 2010 to 2019 at the Chinese PLA General Hospital. Their biopsy tissues were collected at the time of pre-treatment staging.

Patients had to fulfill the following eligibility criteria: (a) completion of diagnosis and treatment process in a single center with available pre-treatment FFPE biopsies; (b) histologically confirmed adenocarcinoma; and (c) completion of 5-FU-based nCRT followed by TME surgery as the first therapeutic approach. Initial clinical staging was based on rectoscopy, thorax-abdomen computed tomography (CT) scan and/or pelvic magnetic resonance imaging (MRI). All patients were assigned to pelvic long-course radiotherapy (50 Gy in 25 fractions over 5 weeks of three-dimensional conformal radiotherapy, 2 Gy per fraction, per day) with concurrent 5-FU-based chemotherapy. Standardized surgery (including abdominoperineal resection; anterior resection; low anterior resection; Hartmann’s operation) was performed after an interval of 6 to 14 weeks post nCRT. The study was approved by the Medical Ethics Committee of the Chinese PLA General Hospital (No. S2021-129-01).

### Collection of clinical data

Clinical data collected from the medical records of patients are detailed in Tables 1 and 2, including age at diagnosis, gender, histological features (grade of differentiation and mucinous histology), and the tumor location (the distance from the anal verge (AV) to the lowest margin of the tumor on either the MRI or rectoscopy), blood routine indices and the levels of tumor biomarkers (detected before nCRT), etc. Pathologic results were reported according to the 8th AJCC TNM staging classification system. Treatment responses to nCRT and tumor regression grade (TRG) were evaluated by experienced pathologists in accordance with the AJCC TRG system [14]. According to the TRG, patients were divided into three groups: total responders (TR: AJCC TRG0), moderate responders (MR: AJCC TRG1), and poor responders (PR: AJCC TRG2+3). To screen out proteins most related to treatment reaction, the TR and PR groups were defined as the internal discovery cohort used for differential expression analysis, while all of the TR, MR, and PR groups were defined as the total internal cohort.

**Table 1:** Clinical baseline characteristics in different response groups

Characteristics of patients		All	TR	PR	*p*
n		20	9	11	
Age (years), Mean ± SD		53.40 ± 10.67	53.89 ± 11.98	53.00 ± 10.05	0.859
Gender, n (%)	Female	11 (55.0)	6 (66.7)	3 (27.3)	0.175
	Male	9 (45.0)	3 (33.3)	8 (72.7)	
Diabetes, n (%)		3 (15.0)	2 (22.2)	1 (9.1)	0.566
Arterial hypertension, n (%)		3 (15.0)	2 (22.2)	1 (9.1)	0.566
Family history of cancer, n (%)		2 (10.0)	0 (0.0)	2 (18.2)	0.236
Body mass index (kg/m^2)^		23.68 ± 3.59	22.81 ± 1.43	24.39 ± 4.65	0.307
Smokers, n (%)		7 (35.0)	0 (0.0)	7 (63.6)	0.005
Anemia, n (%)		2 (10.0)	1 (11.1)	1 (9.1)	1.000
Tumor localization, n (%)	Inferior rectum (< 5 cm)	7 (35.0)	3 (33.3)	4 (36.4)	0.621
	Mid rectum (5–10 cm)	12 (60.0)	6 (66.7)	6 (54.5)	
	Superior rectum (> 10 cm^)^	1 (5.0)	0 (0.0)	1 (9.1)	
MRI	Tumor diameter (mm)	40.50 ± 13.66	43.00 ± 7.35	50.20 ± 16.88	0.247
	Initial lateral lymph node dissemination, n (%)	10 (50.0)	5 (55.6)	5 (45.5)	1.000
cTNM, n (%)	II	6 (30.0)	2 (22.2)	4 (36.4)	0.850
	III	10 (50.0)	5 (55.6)	5 (45.5)	
	Missing data	4 (20.0)	2 (22.2)	2 (18.2)	
nCRT time (days), Median (Q1-Q3)		35 (33–35)	35 (34–39)	34 (32–35)	0.110
Surgery delay (days), Median (Q1-Q3)		55 (47–63)	60 (50–63)	48 (46–75)	0.381
Anus preservation, n (%)		11 (55.0)	6 (66.7)	5 (45.5)	0.406
R0 resection, n (%)		20 (100.0)	9 (100.0)	11 (100.0)	/

*P* values for TR vs. PR. TR, total responders. PR, poor responders. nCRT, neoadjuvant chemoradiation therapy. MRI, magnetic resonance imaging

**Table 2:** Associations of pathological characteristics with different responses to nCRT

Pathological characteristics		All	TR	PR	*p*
n		20	9	11	
Histological classification (biopsy), n (%)	Moderately differentiated	19 (95.0)	8 (88.9)	11 (100.0)	0.450
	Not available	1 (5.0)	1 (11.1)	0 (0.0)	
Mucinous classification, n (%)	Non-mucinous	19 (95.0)	8 (88.9)	11 (100.0)	0.450
	Mucinous	1 (5.0)	1 (11.1)	0 (0.0)	
Neurovascular invasion, n (%)		2 (10.0)	0 (0.0)	2 (18.2)	0.479
ypStage, n (%)	pCR	9 (45.0)	9 (100.0)	0 (0.0)	0.000
	I	2 (10.0)	0 (0.0)	2 (18.2)	
	II	6 (30.0)	0 (0.0)	6 (54.5)	
	III	3 (15.0)	0 (0.0)	3 (27.3)	
Pathological T (TNM system), n (%)	T0	9 (45.0)	9 (100.0)	0 (0.0)	< 0.001
	T2	3 (15.0)	0 (0.0)	3 (27.3)	
	T3	7 (35.0)	0 (0.0)	7 (63.6)	
	T4	1 (5.0)	0 (0.0)	1 (9.1)	
Pathological N (TNM system), n (%)	N0	17 (85.0)	9 (100.0)	8 (72.7)	0.236
	N1	2 (10.0)	0 (0.0)	2 (18.2)	
	N2	1 (5.0)	0 (0.0)	1 (9.1)	
ypT_downstage, n (%)	Yes	10 (50.0)	8 (88.9)	2 (18.2)	0.001
	No	7 (35.0)	0 (0.0)	7 (63.6)	
	Missing data	3 (15.0)	1 (11.1)	2 (18.2)	
ypN_downstage, n (%)	Yes	11 (55.0)	6 (66.7)	5 (45.5)	0.552
	No	7 (35.0)	2 (22.2)	5 (45.5)	
	Missing data	2 (10.0)	1 (11.1)	1 (9.1)	

*P* values for TR vs. PR.TR, total responders. PR, poor responders. nCRT, neoadjuvant chemoradiation therapy

### Protein extraction

Proteins from FFPE samples were extracted using the FFPE Total Protein Extraction Kit (Sangon Biotech, Shanghai, China) following the manufacturer's instruction. Then samples were precipitated with 4 × (v/v) ice-cold acetone and incubated at – 20 ℃ overnight. The precipitated proteins were centrifuged at 10,000×*g* at 4℃ for 15 min and air-dried.

### Proteomic analysis

Samples were digested following the filter-aided sample preparation (FASP) method with Amicon Ultra-0.5 centrifugal filters (Merck Millipore, Darmstadt, Germany) with 10 kDa MW cut-off. In detail, protein samples were dissolved in 8 M urea. 100 μg protein was reduced with 10 mM dithiothreitol (DTT) at 37 ℃ for 120 min and alkylated with 20 mM iodoacetamide (IAA) in the same solvent (23 ℃ for 30 min, in darkness), followed by a three-washes with 50 mM ammonium bicarbonate and digestion with 2 μg trypsin (V5111; Promega, Madison, WI, USA) overnight at 37 ℃. Post vacuum drying, peptides were reconstituted in 0.1% formic acid for MS analysis.

LC–MS/MS analysis of tryptic peptides was performed on a quadrupole Orbitrap mass spectrometer (Q Exactive, Thermo Fisher Scientific, Waltham, MA, USA) coupled to Dionex Ultimate-3000 HPLC system (Thermo Fisher Scientific) via a nano-electrospray ion source. The peptides (~ 350 ng) of peptides were separated using an in-house packed C18 analytical column (Magic C18 particles, 3 μm, Michrom Bioresource, Auburn, CA, USA) and measured over a total gradient length of 120 min with increasing buffer B (80% acetonitrile [ACN] and 0.08% formic acid; Merck, Darmstadt, Germany) concentration. The mass spectrometer was operated in data-dependent acquisition (DDA) mode.

### Analysis of MS data

Q-Exactive raw data were searched against the uniprot_sprot.fasta database of Homo sapiens protein sequence and processed to calculate the area for each protein using Proteome Discoverer (PD) software v2.1.1.21 (Thermo Scientific).

For a more in-depth statistical analysis, the area data were then processed using Perseus v1.6.15.0 (freeware, Max Planck Institute of Biochemistry). Normalized and log2-transformed signal intensity values were used for subsequent analyses. Differentially expressed proteins (DEPs) were defined as proteins with a fold change > 1.5 between the TR and PR groups and a *p* < 0.05 (Student's *t*-test). Volcano plots, heatmaps, and partial least squares discriminant analysis (PLS-DA) were performed using pre-processed data with the online tools MetaboAnalyst 5.0 (https://www.metaboanalyst.ca) and Hiplot (https://hiplot.com.cn).

### External validation

External validation was performed with the ROC plotter (www.rocplot.org) [15], an online database that links gene expression and response to therapy using transcriptome-level data of patients with RC. Within the database, a total of 56 patients with RC treated with radiotherapy and capecitabine were grouped into responders (n = 15) and non-responders (n = 41) according to the Response Evaluation Criteria in Solid Tumors (RECIST) criteria. The levels of probes corresponding to the DEPs of the internal discovery cohort in the responder and non-responder groups were compared, and probes with significant difference between the two groups were further analyzed using the ROC curve so as to evaluate their predictive performance.

### GSCALite

GSCALite (http://bioinfo.life.hust.edu.cn/web/GSCALite/) [16] was used to analyze the relationship between genes and pathways by a line connection, as well as the correlation of gene expression and 5-FU sensitivity based on the Cancer Therapeutics Response Portal (CTRP) and Genomics of Drug Sensitivity in Cancer (GDSC) drug response datasets. The effects of genes on activation or inhibition of cancer-related pathways in specific type of cancer were evaluated using reverse phase protein array (RPPA) data from The Cancer Proteome Atlas (TCPA). The following pathways which are closely related to cancer therapy resistance were explored: epithelial mesenchymal transition (EMT), cell cycle, apoptosis pathways.

### Bioinformatic analysis

To explore the possible underlying mechanism, functional gene set enrichment analysis (GSEA) was performed on the whole proteome data of the internal discovery cohort using Hallmark gene sets with default parameters. The GSEA results were filtered based on a nominal (NOM) *p*-value < 0.01 and the Normalized Enrichment Scores (NES) was used to identify top-ranked gene sets in TR versus PR. Then, for the DEPs, Gene Ontology (GO) and Kyoto Encyclopedia of Genes and Genomes (KEGG) enrichment were performed using DAVID Bioinformatics Resources v6.8 (https://david.ncifcrf.gov/home.jsp) and visualized using Barplot (v0.1.1) in Hiplot. Pathways with *p* < 0.05 were considered significantly enriched. The protein–protein interaction (PPI) network was assessed using the STRING database (https://string-db.org/) and visualized by Cytoscape v3.8.2[17]. The DEGs were ranked using the ‘cytoHubba’ plugin of Cytoscape and displayed with color transition.

### Statistical analysis

Statistical analysis was performed using Graphpad Prism v7.0 (GraphPad, San Diego, CA, USA). Statistical comparisons between the TR and PR groups were performed by Student *t*-test, Manny-Whitney *U*-test or chi-square test as appropriate. To investigate the linear relationship between each protein level and therapeutic responses, the *p*-value for trend was calculated by treating TR, MR, and PR groups as continuous variables. Correlation analysis between potential biomarkers and clinical features was performed using Spearman's coefficient of correlation. To evaluate the discrimination performance of the protein levels between different groups, the ROC curve analysis was conducted, and the true positive rate (TPR), true negative rate (TNR), area under the curve (AUC) were calculated. Statistical significance was set at *p* < 0.05.

## Results

### Clinical and pathological features of patients with LARC

**Fig. 1:**
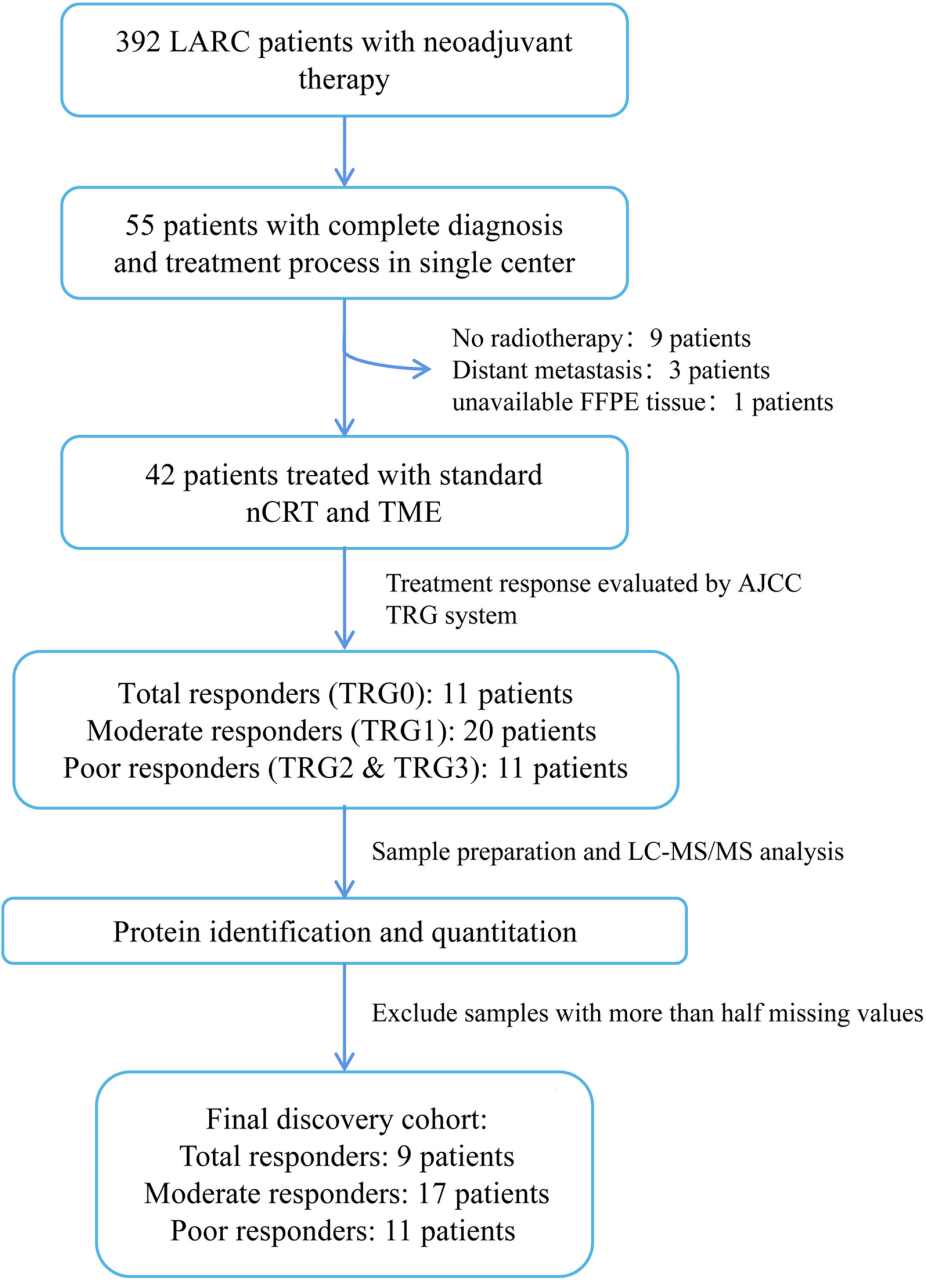
Workflow of the total internal cohort in Chinese PLA General Hospital from 2010 to 2019. A total of 42 patients with LARC met the enrollment criteria and were divided into 3 groups based on TRG, namely total responders (TR), moderate responders (MR), and poor responders (PR). Samples from 37 patients passing quality control in protein identification were finally included in the discovery cohort for further data analysis. LARC, locally advanced rectal cancer; nCRT, neoadjuvant chemoradiation therapy; TME, total mesorectal excision; TRG, tumor regression grade

Among 392 patients with LARC treated with neoadjuvant therapy from the Chinese PLA General Hospital from 2010 to 2019, 42 patients were enrolled according to the criteria. Eventually, 37 patients were confirmed in the total internal cohort after excluding 5 samples with more than half missing values found in the process of protein identification and quantitation (Fig. 1). The cases in the total responders (TR), moderate responders (MR) and poor responders (PR) groups were 9 (24.32%), 17 (45.95%) and 11 (29.73%), respectively. The TR and PR groups were selected for further analysis of differentially expressed proteins (DEPs). Most patients in the TR and PR groups were aged over 53 years with mid to inferior rectal tumor (95%), non-mucinous adenocarcinoma (95%) disease.

Clinical and pathological features of patients with LARC were detailed in Tables 1 and 2. There were no statistical differences between the TR and PR groups in age, gender, body mass index (BMI), chronic complications, tumor location and size, initial lateral lymph node dissemination, clinical TNM stage, nCRT time, or pathological related characteristics.

Results of hematology tests and serum tumor markers (CEA, CA 19-9, CA 125, and AFP) detected at the time of diagnosis before nCRT were also obtained, but no significant differences were observed between the TR and PR groups (Table 3).

**Table 3:** Baseline comparison of blood routine and tumor biomarkers in different responses to nCRT from the internal discovery cohort

Laboratory index	TR	PR	*p*
Haemoglobin (g/L)	130.29 ± 17.67	133.30 ± 13.43	0.694
RBC (10^12^/L)	4.46 ± 0.46	4.52 ± 0.46	0.790
WBC (10^9^/L)	5.80 ± 2.08	5.91 ± 2.00	0.907
Neutrophil	0.63 ± 0.07	0.62 ± 0.10	0.963
Lymphocyte	0.30 ± 0.07	0.28 ± 0.07	0.492
Monocyte	0.06 ± 0.014	0.06 ± 0.018	0.669
Eosinophil	0.01 ± 0.005	0.02 ± 0.017	0.169
Basophil	0.003 ± 0.002	0.004 ± 0.003	0.421
Platelet (10^9^/L)	252 ± 58	200 ± 98	0.231
NLR	2.22 ± 0.77	2.48 ± 1.02	0.587
Fibrinogen (g/L)	3.38 ± 0.74	3.47 ± 0.47	0.764
CEA (μg/L)	5.50 ± 4.45	8.08 ± 8.61	0.519
AFP (μg/L)	3.17 ± 2.45	4.51 ± 3.55	0.445
CA125 (U/mL)	19.44 ± 13.49	11.46 ± 3.94	0.194
CA19-9 (U/mL)	43.35 ± 48.64	17.15 ± 19.78	0.184

*TR* total responders, *PR* poor responders, *RBC* Red blood cell, *WBC* White blood cell, *NLR* Neutrophil/Lymphocyte, *CEA* carcinoembryonic antigen, *AFP* alpha-fetoprotein, *CA125* Carbohydrate antigen 125, *CA199* Carbohydrate antigen 19-9

### Differentially expressed proteins between TR and PR group

**Fig. 2:**
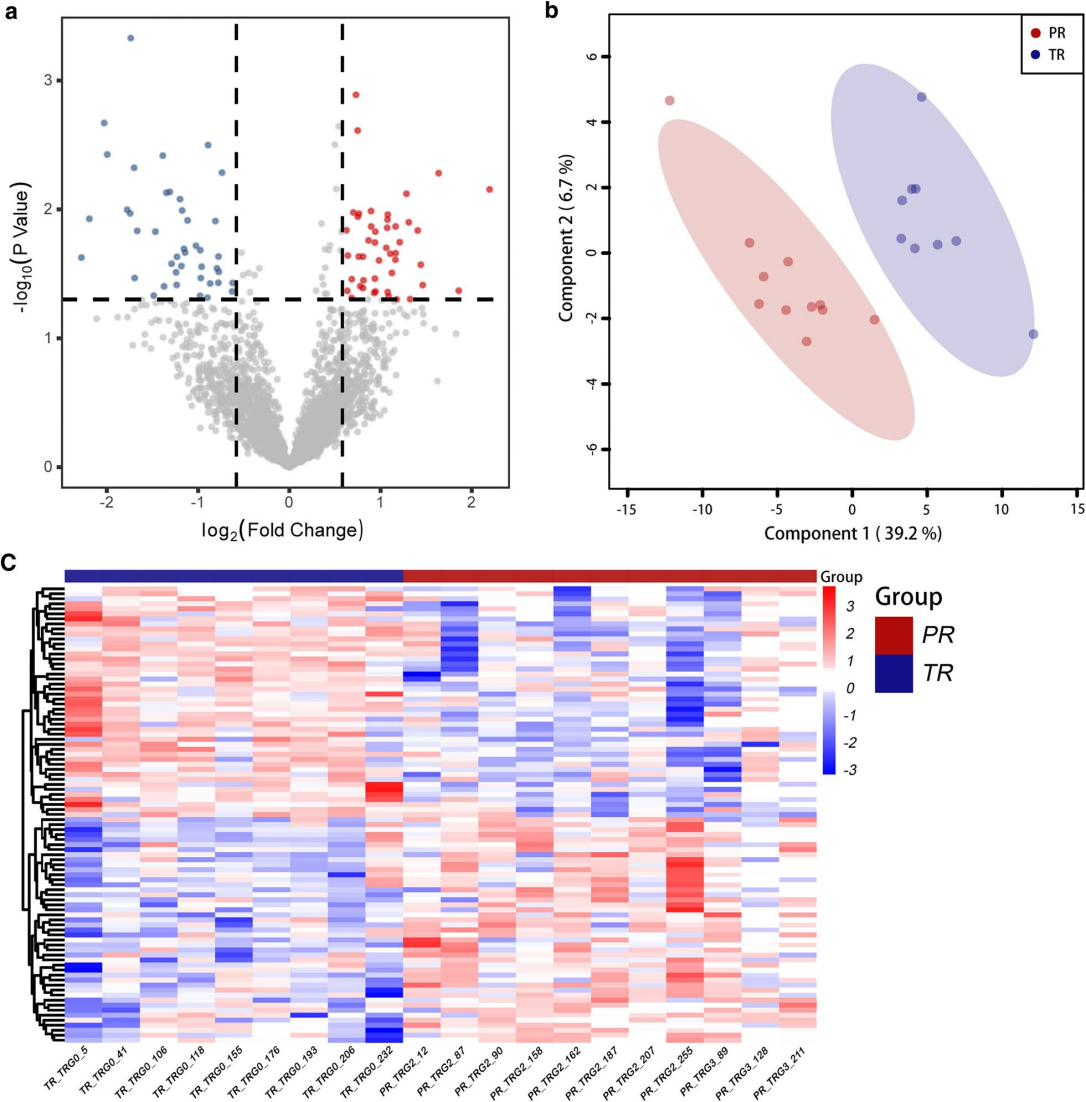
Differential protein expression characteristics. **a** Volcano plots show the distribution of quantified proteins according to -log_10_ (*p*-value) and log_2_ (fold-change) of mean LFQ intensity difference. **b** PLS-DA plots show significant separation and discrimination between the TR and PR groups. **c** Differentially expressed proteins (fold-change ≥ 1.5) are colored based on the heat map scale (red: upregulated in TR group, blue: downregulated in TR group). TR, total responders; PR, poor responders

In total, 3,998 proteins were identified through LC–MS/MS-based proteomic analyses on FFPE biopsy samples from patients with LARC. As visualized in the volcano plot (Fig. 2a) and heatmap (Fig. 2c), 91 DEPs were found with statistical significance (*p* < 0.05) between the TR and PR group, including 45 up- and 46 downregulated proteins in the PR group compared with the TR group (Additional file 1: Table S1 and Additional file 2: Table S2). PLS-DA plot (Fig. 2b) shows that these DEPs levels were distinguished and clustered between the TR and PR groups.

### Validation of potential biomarkers for response to nCRT

**Fig. 3:**
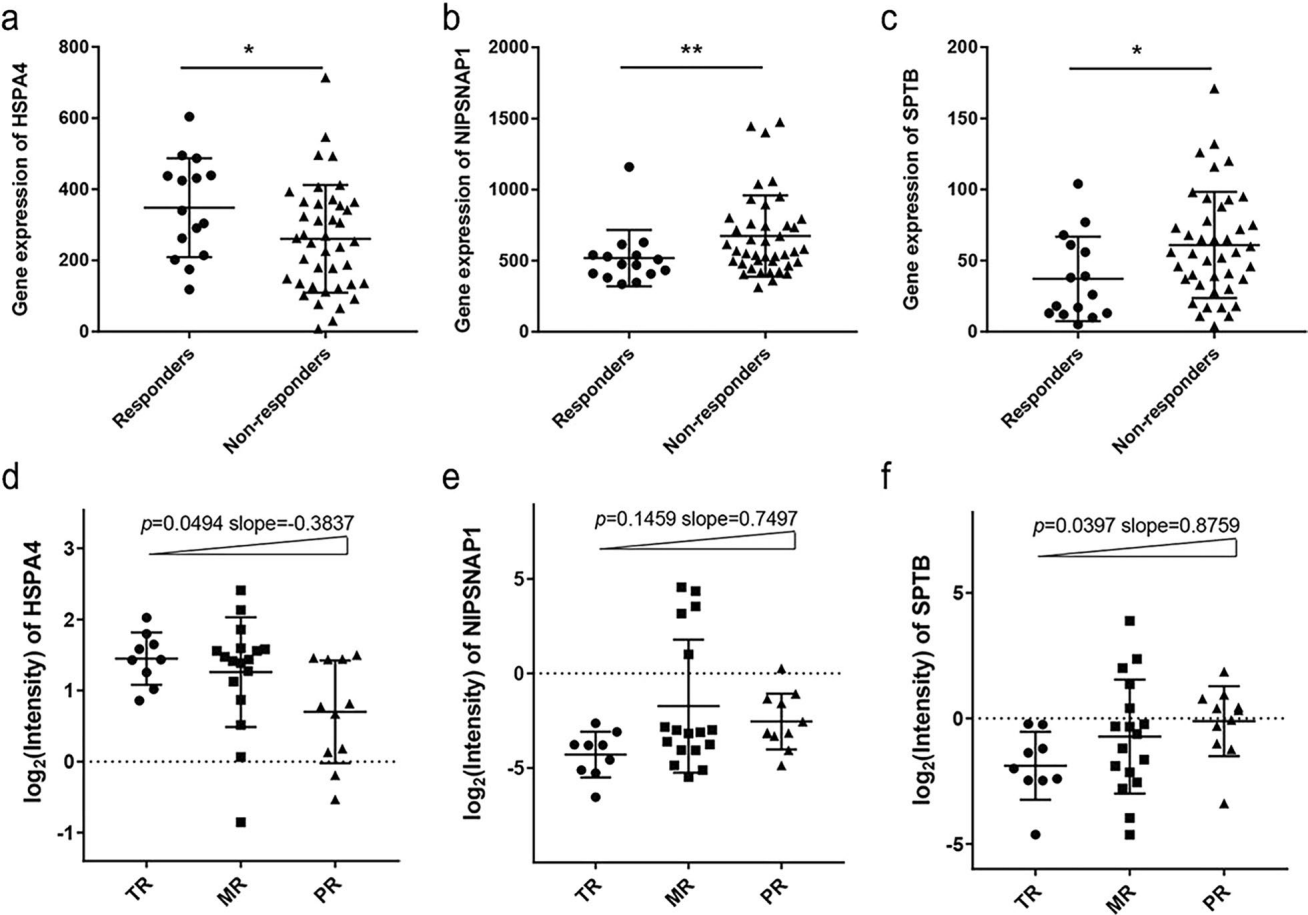
Validation of the proteins associated with 5-FU-based nCRT resisitance. **a**–**c** The gene expression in the external validation cohort (n = 56) is shown as scatter plots. Statistical significance between responders and non-responders was calculated using the Mann–Whitney test. **d**–**f** The relationship between the individual protein level of HSPA4, NIPSNAP1, and SPTB, as well as the therapeutic response grades in the total internal cohort (n = 37), are shown as scatter plots. *P*-values for trends were calculated using the one-way ANOVA post-test for linear trends. TR, total responders; MR, moderate responders; PR, poor responders. (*p < 0.05; **p < 0.01 and ***p < 0.001)

To assess the predictive value of the DEPs for nCRT response in patients with RC, an online ROC Plotter tool was utilized. From the ROC plotter, a total of 56 patients with RC receiving treatments of radiotherapy and capecitabine, as the external validation cohort, were divided into non-responders (n = 41) and responders (n = 15) according to the RECIST criteria. Based on the gene expression data of the external cohort, expression levels of heat shock proteins family A member 4 (*HSPA4)*, nitrophenylphosphatase domain and non-neuronal SNAP25-like protein homolog 1 (*NIPSNAP1)* and spectrin beta, erythrocytic (*SPTB)* were validated to be statistically different between the responders and non-responders (*p* = 0.026, *p* = 0.009, *p* = 0.013, respectively) with higher expression of *HSPA4* and lower expressions of *NIPSNAP1* and *SPTB* in the responders (Fig. 3a–c), which were in accordance with our findings from the internal discovery cohort.

Then, we further explored the variation tendencies of HSPA4, NIPSNAP1 and SPTB among different response groups in the total internal cohort, and we had confirmed the dose-dependent relationship between each of the three proteins and therapy response grades. Significant linear trends were found across the TR, MR and PR groups in terms of HSPA4 (*p* = 0.0494, slope = 0.3837) and SPTB (*p* = 0.0397, slope = 0.8759) (Fig. 3d, f), indicating that the levels of HSPA4 and SPTB varied with the extent of nCRT response. Although not statistically different, a seemingly linear trend in the NIPSNAP1 level among these response groups was observed (*p* = 0.1459, slope = 0.7497) (Fig. 3e).

### The relationship between the clinical features and the expressions of HSPA4, NIPSNAP1, and SPTB

The correlation analysis were performed in the total internal cohort to explore the relationship of the disease stage, as well as smoking status, with the expressions of HSPA4, NIPSNAP1, and SPTB. No significant correlation was found between cTNM staging and the expression of HSPA4 (Spearman correlation coefficient: 0.298, *p* = 0.123), NIPSNAP1 (Spearman correlation coefficient: − 0.166, *p* = 0.399), SPTB (Spearman correlation coefficient: 0.109, *p* = 0.581), respectively. Also, there was no significant correlation between smoking status and the expression of HSPA4 (Spearman correlation coefficient: − 0.168, *p* = 0.321), NIPSNAP1 (Spearman correlation coefficient: 0.234, *p* = 0.164), SPTB (Spearman correlation coefficient: 0.112, *p* = 0.510), respectively.

### Evaluation of HSPA4, NIPSNAP1, and SPTB as predictive markers for nCRT response

**Fig. 4:**
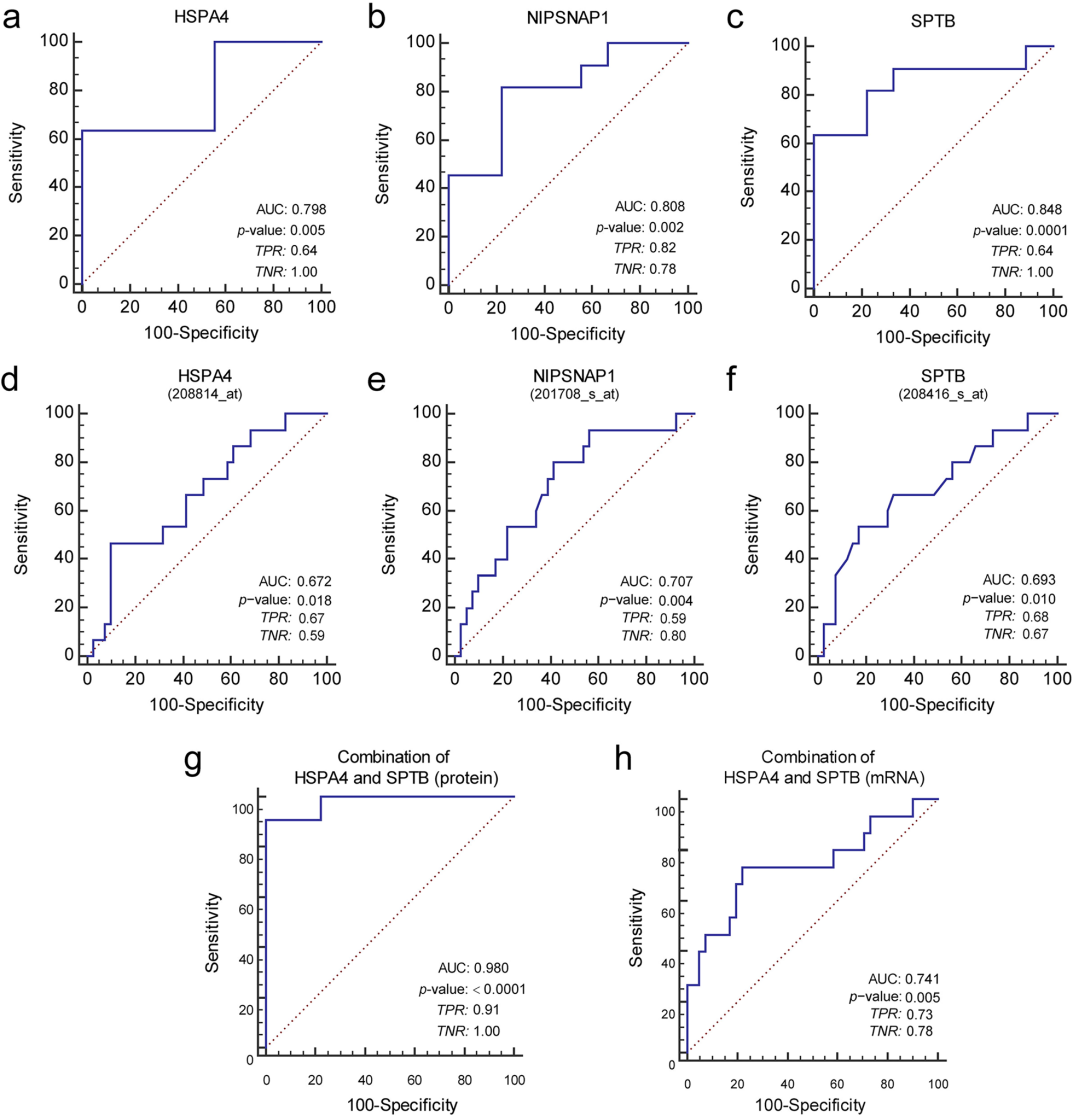
ROC curves of HSPA4, NIPSNAP1, SPTB, as well as the combination of HSPA4 and SPTB. ROC curves of HSPA4, NIPSNAP1, SPTB, as well as the combination of HSPA4 and SPTB for predictive performance in the internal discovery cohort (n = 20; total responders = 9, poor responders = 11) (**a**–**c**, **g**) and the external validation cohort (n = 56, responders = 15, non-responders = 41) (**d**–**f**, **h**). ROC, receiver operating characteristic;. AUC, area under the curve; TPR, true positive rate; TNR, true negative rate

As significant linear trends were discovered in the levels of HSPA4 and SPTB across various responses to nCRT in patients with RC, the predictive performance of these potential markers in the TR and PR groups was assessed using ROC curve analysis. Detailed results are listed in Table 4. The three proteins had individual AUCs of ~ 0.8 in the internal discovery cohort (Fig. 4a–c) and ~ 0.7 in the external validation cohort from the ROC plotter database (Fig. 4d–f). Intriguingly, the AUC of HSPA4 combined with SPTB dramatically increased to 0.980 (95% CI, 0.797–1.000, *p* < 0.0001) in the discovery cohort, with sensitivity and specificity of 90.91 and 100.00%, respectively (Fig. 4g). In the external validation cohort, the combination of *HSAP4* and *SPTB* mRNA achieved an AUC of 0.741 (95% CI 0.607–0.849, *p* = 0.0048), with sensitivity and specificity of 73.33 and 78.05%, respectively (Fig. 4h).

**Table 4:** AUCs of potential protein markers and their combinations in the internal discovery cohort and external validation cohort

Group	Marker	AUC	SE	TNR	TPR	*p* value	95% CI of AUC	
							Lower bound	Upper bound
Internal discovery cohort	HSPA4	0.798	0.103	1.00	0.64	0.004	0.561	0.942
	NIPSNAP1	0.808	0.099	0.78	0.82	0.0018	0.572	0.947
	SPTB	0.848	0.091	1.00	0.64	0.0001	0.619	0.967
	Combination 1	0.939	0.051	1.00	0.82	< 0.0001	0.736	0.997
	Combination 2	0.980	0.024	1.00	0.91	< 0.0001	0.797	1.000
	Combination 3	0.859	0.096	0.78	0.91	0.0002	0.631	0.972
External validation cohort	HSPA4	0.672	0.082	0.90	0.47	0.036	0.533	0.791
	NIPSNAP1	0.707	0.078	0.59	0.80	0.008	0.570	0.821
	SPTB	0.693	0.084	0.83	0.53	0.021	0.556	0.810
	Combination 1	0.735	0.070	0.51	0.93	0.0008	0.600	0.844
	Combination 2	0.741	0.086	0.78	0.73	0.005	0.607	0.849
	Combination 3	0.732	0.082	0.90	0.53	0.005	0.597	0.841
	Combination 4	0.780	0.076	0.88	0.67	0.0002	0.650	0.880

*ROC* receiver operating characteristic, *AUC* area under the curve, *TPR* true positive rate, *TNR*, true negative rate. Combination 1: HSPA4 & NIPSNAP1; Combination2: HSPA4 & SPTB; Combination 3: NIPSNAP1 & SPTB; Combination 4: HSPA4 & NIPSNAP1 & SPTB

Next, the MR group was introduced into the TR and PR groups, respectively, for ROC analysis to observe the predictive performance of HSPA4, NIPSNAP1, and SPTB, along with their combinations. As shown in Table 5, for TR versus MR & PR, the highest TPR reached up to 0.79 with AUC = 0.849 (*p* < 0.0001) when HSPA4 and SPTB were combined (Combination 2), which meant Combination 2 had the best identification ability for the total responders. For TR&MR versus PR results, the highest TNR reached 0.97 combining HSPA4, NIPSNAP1, and SPTB (Combination 4, AUC = 0.794, *p* = 0.0007), meaning that Combination 4 best identifid poor responders compared with others.

**Table 5:** AUCs of potential protein markers and their combinations in the total internal cohort

Group	Marker	AUC	SE	TNR	TPR	*p* value	95% CI of AUC	
							Lower bound	Upper bound
TR versus MR + PR	HSPA4	0.647	0.102	1.00	0.36	0.148	0.473	0.796
	NIPSNAP1	0.762	0.090	0.78	0.75	0.004	0.594	0.886
	SPTB	0.722	0.087	0.78	0.68	0.011	0.551	0.856
	Combination 1	0.762	0.090	0.78	0.75	0.004	0.594	0.886
	Combination 2	0.849	0.064	0.89	0.79	< 0.0001	0.693	0.945
	Combination 3	0.869	0.063	1.00	0.61	< 0.0001	0.717	0.957
	Combination 4	0.897	0.055	1.00	0.58	< 0.0001	0.752	0.972
TR + MR versus PR	HSPA4	0.748	0.089	0.88	0.64	0.006	0.579	0.876
	NIPSNAP1	0.615	0.097	0.54	0.82	0.233	0.441	0.770
	SPTB	0.703	0.095	0.81	0.64	0.032	0.530	0.841
	Combination 1	0.689	0.107	0.77	0.64	0.078	0.516	0.830
	Combination 2	0.836	0.069	0.69	0.91	< 0.0001	0.677	0.937
	Combination 3	0.710	0.095	0.73	0.73	0.027	0.538	0.847
	Combination 4	0.794	0.087	0.97	0.56	0.0007	0.629	0.909

*ROC* receiver operating characteristic, *AUC* area under the curve, *TPR* true positive rate, *TNR* true negative rate, *TR* total responders, *MR* moderate responders, *PR* poor responders. Combination 1: HSPA4 & NIPSNAP1; Combination2: HSPA4 & SPTB; Combination 3: NIPSNAP1 & SPTB; Combination 4: HSPA4 & NIPSNAP1 & SPTB

### Correlation of the DEPs with drug sensitivity database

**Fig. 5:**
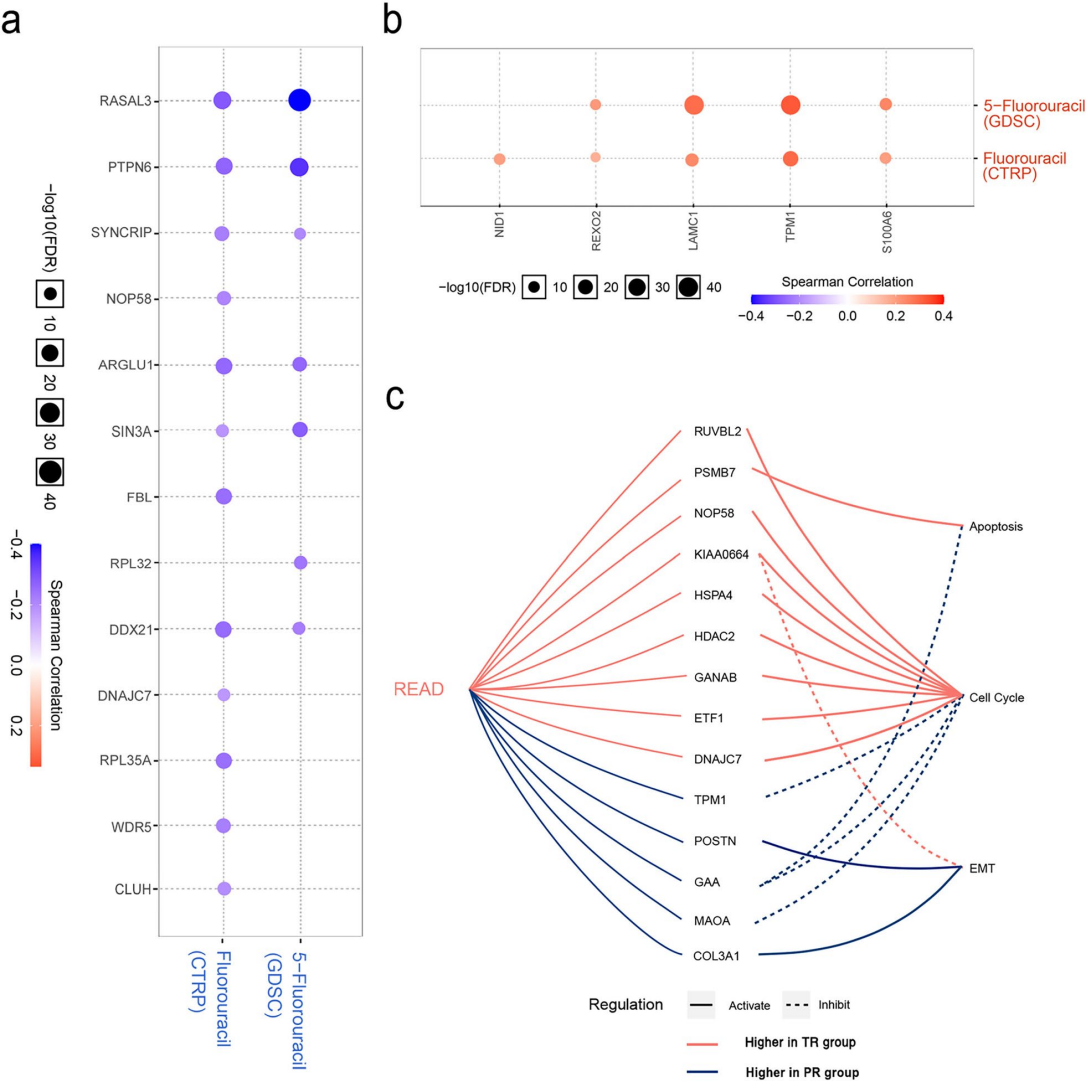
Relationship between genes/proteins and drug sensitivity/pathway activities in rectal cancer. **a**, **b** 5-FU resistance analysis of the differentially expressed proteins (DEPs) corresponding to genes based on GDSC/CTRP IC_50_ drug data. Gene expression correlation with the drug sensitivity was determined using Spearman correlation. A positive correlation means the genes with high expression are resistant to the drug (fluorouracil or 5-fluorouracil), and vise verse. **c** The role of the DEPs in cancer related-pathways (using GSCALite). Reverse-phase protein array data from The Cancer Proteome Atlas are analyzed to calculate the correlation of genes with cancer-related pathways in rectal cancer. Genes corresponding to the DEPs with activation/inhibition effect on apoptosis, cell cycle and EMT pathways are shown. TR, total responders; PR, poor responders; EMT, epithelial mesenchymal transition; READ, rectal adenocarcinoma

Based on the drug sensitivity data of the cancer cell lines in the CTRP and GDSC databases, the corresponding genes of the DEPs were validated for their correlation with 5-FU sensitivity in RC using the online GSCALite website tool. The results revealed that *RASAL3*, *PTPN6*, *SYNCRIP*, *ARGLU1*, *SIN3A* and *DDX21* had higher expression in total responders which negatively correlated to 5-FU resistance (Fig. 5a), while *REXO2*, *LAMC1*, *TPM1* and *S100A6* had lower expression in the total responders showing positive correlation with 5-FU resistance (Fig. 5b).

### Effects of the DEPs on oncogenic pathways in RC

To further understand the molecular mechanisms for the DEPs involved in tumorigenesis of RC, the GSCALite tool was used to examine the correlation between gene expression levels and regulation of three key signaling pathways which were closely related to therapeutic response, according to a pathway score calculated by GSCALite. Our results showed that the DEPs were highly associated with the activation or inhibition of apoptosis, cell cycle, and EMT pathways (Fig. 5c). Concerning cell cycle pathways, HSPA4, RUVBL2, NOP58, CLUH, HDAC2, GANAB, ETF1, and DNAJC7 were higher in the TR group and closely related to the activation of the cell cycle, while TPM1, GAA, and MAOA were found higher in the PR group and associated with the inhibition of the cell cycle. In addition, the apoptosis pathway could be activated by PSMB7 and inhibited by GAA. Lastly, POSTN and COL3A1 had higher levels in the PR group involved in activation of the EMT pathway.

### Functional enrichment and PPI networks

**Fig. 6:**
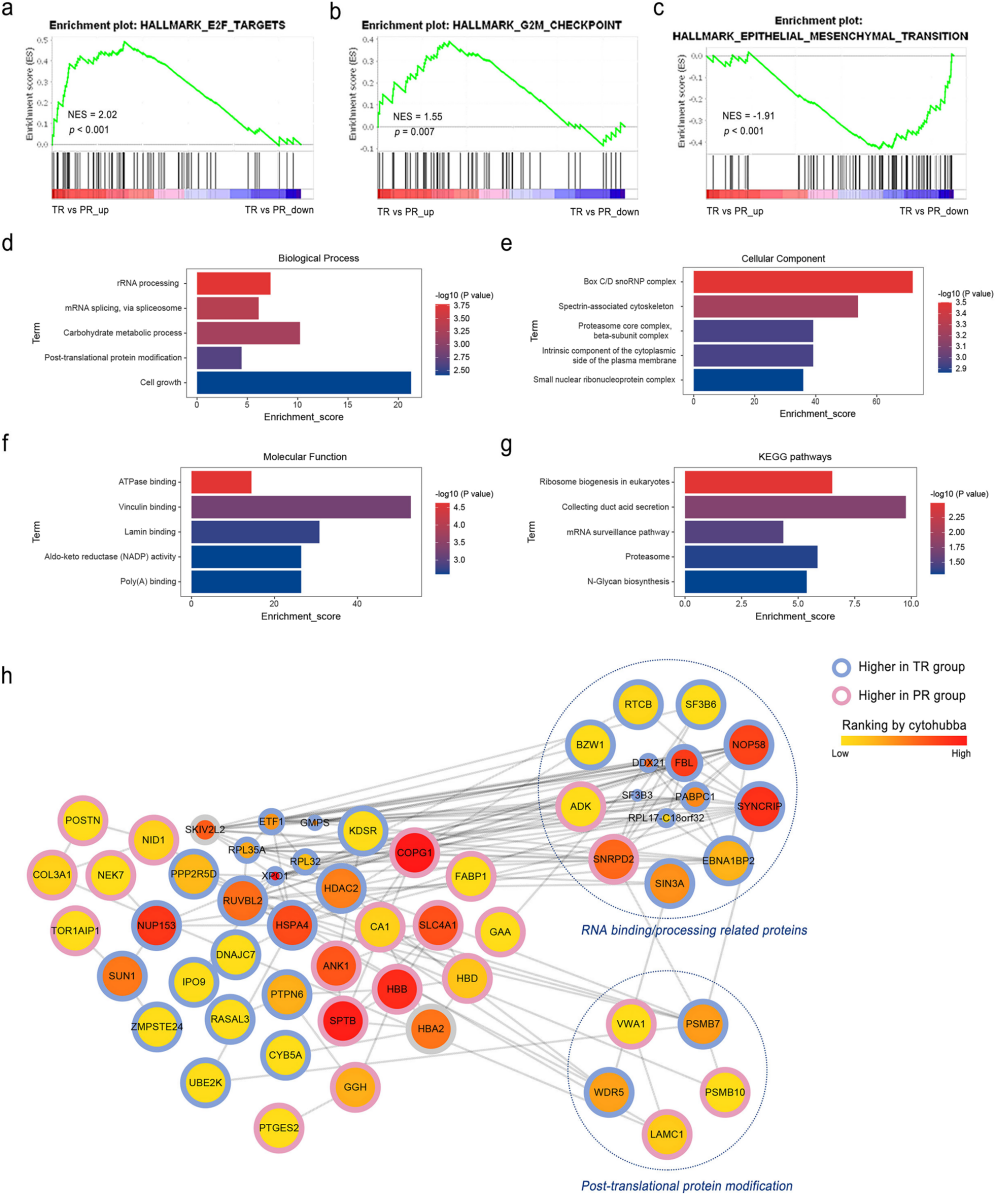
Functional enrichment and protein–protein interaction (PPI). Gene set enrichment analysis (GSEA) results of the whole proteomic data. HALLMARK gene set terms “E2F_TARGETS” (**a**) and “G2/M_CHECKPOINT” (**b**) significantly (nominal *p* < 0.01) upregulated (red) and “EPITHELIAL_MESENCHYMAL_TRANSITION” (**c**) downregulated (blue) in the TR versus PR group. The top five Gene Ontology (GO) analysis terms in the biological process (**d**), cellular component (**e**), and molecular function (**f**) categories of differentially expressed proteins between the TR and PR group. **g** The differentially expressed proteins were mapped to canonical pathways using the Kyoto Encyclopedia of Genes and Genomes (KEGG) enrichment. **h** The PPI network was assessed using the STRING database and visualized by Cytoscape. Color transition of the nodes is based on the ranking calculated by CytoHubba. NES, Normalized Enrichment Scores; TR, total responders; PR, poor responders

Through the GSEA results, proteomics revealed that the TR group was enriched in gene sets related to cell cycle such as the “E2F targets” (NES = 2.02, *p* < 0.001) and “G2M checkpoint” (NES = 1.55, *p* = 0.007), while the PR group was enriched in EMT (NES = 1.91, *p* < 0.001) (Fig. 6a–c). Then, GO and KEGG enrichment analysis gave further insight into the biological functions of the DEPs. The top five terms of GO and KEGG annotations showed the DEPs were mainly associated with processes including rRNA processing, Box C/D snoRNP complex, ATPase binding and ribosome biogenesis in eukaryotes (Fig. 6d–g). As shown in the PPI network (Fig. 6h), 14 proteins were involved in RNA binding or processing processes, of which 12 proteins were higher in the TR group. The 10 most connected nodes ranked (in descending order) by cytoHubba were XPO1, COPG1, SPTB, SF3B3, HBB, SYNCRIP, NUP153, FBL, NOP58, and HSPA4.

## Discussion

nCRT before TME is the standard treatment for patients with LARC, unfortunately, various patients could not achieve favorable responses to nCRT. To avoid futile treatments for non-responders, effective prediction of nCRT response would guide treatment decisions for patients with LARC, while also increasing the opportunity for responders to receive nCRT and perform organ-conserving surgery. Various studies have been conducted to identify predictive markers for response to nCRT in patients with LARC using proteomic methods. However, to the best of our knowledge, the present study was the first to identify proteins that could discriminate between good and poor responders in Chinese patients with LARC.

Through proteomic approach, 91 DEPs were screened from different nCRT response groups in FFPE biopsy tissues of patients with LARC to successfully discriminate total and poor responders by PLS-DA analysis. Further verification demonstrated the promising discrimination ability of HSPA4, NIPSNAP1, and SPTB for different nCRT responses in both internal and external cohorts. Significant linear trends found in HSPA4 and SPTB among the TR, MR, and PR groups revealed changing levels to therapeutic responses, which implied their potential predictive value for nCRT response.

Subsequently, we utilized ROC analysis to evaluate the predictive performance of the three proteins. Though each of them showed moderate performance in both cohorts, the AUCs of their various combinations distinctly improved discrimination between the total and poor responders, especially the combination of HSPA4 and SPTB whose AUC surprisingly reached 0.98 in our cohort. In clinical applications, a greater concern is placed on one marker that could accurately discriminate between total responders and poor responders in patients with LARC. Therefore, the MR group was further introduced into the ROC analysis to evaluate the predictive value of the three proteins and their different combinations for the entire treatment population. Similarly, the predictive performance of individual proteins and different combinations varied. For TR versus (MR & PR), the combination with the highest sensitivity (HSPA4 & SPTB) was selected so that more patients with total response would be identified and benefit from nCRT. For TR & MR versus PR, the combination with the largest specificity (HSPA4 & NIPSNAP1 & SPTB) was chosen so that patients with poor responses could be mostly identified for personalized their treatment regimens. Therefore, based on the actual requirement, optional combinations of these proteins were determined to improve the discrimination between total response and poor response to nCRT, which provided better guidance on clinical decisions. In summary, our results indicated that the predictive value of multiple combinations of HSPA4, NIPSNAP1, and SPTB were comparable or even superior to predictive markers reported by other studies[18–21], suggesting that they might be reliable predictive markers for nCRT response in patients with LARC.

To explore if the DEPs played a role in 5-FU sensitivity, we analyzed the drug response data from the GDSC and CTRP databases along with related published literatures. Only parts of the DEPs were verified to be associated with the sensitivity or resistance to 5-FU, whereas HSPA4, NIPSNAP1, and SPTB appear to not affect treatment response through 5-FU sensitization. For a better understanding of the roles of the DEPs and the molecular mechanisms underlying the different therapy responses, we next explored the DEP-related processes and pathways. From the GSEA results and the roles of DEPs in cancer related-pathways, we found proteomic differences between the total and poor responders involved in the regulation of the cell cycle and EMT pathways in RC, which were closely related to chemo- and radiotherapy resistance in cancer [22]. DNA damage is one of the major processes induced by both radiation and chemotherapy. In our results, the DEPs were significantly enriched in procedures including RNA binding and processing, and post-translational protein modification. It is known that radiation could cause greater post-translational protein modification with activated intracellular signaling pathways, thus leading to DNA damage. RNA processing might also be associated with the activation of DNA damage response, which in turn or in parallel triggered nucleolar and ribosomal stress [23]. Thus, the DEPs implicated in these processes might affect treatment efficacy via DNA damage and DNA damage repair pathways. Based on our results, we found that HSPA4 is involved in cell cycle activation in RC, while HSPA4 and SPTB were identified as two of the top 10 most connected nodes ranked by cytoHubba. Regrettably, we could not elucidate the machanism of NIPSNAP1 and SPTB, and the roles and underlying mechanism of all three proteins in nCRT response remained unclear.

Heat Shock Proteins (HSPs) are a large family of evolutionally conserved and ubiquitously expressed molecular chaperones. Aberrant levels of HSPs were validated in multiple cancer types and had been shown to be involved in the regulation of malignant progression including apoptosis, proliferation, angiogenesis, metastasis and immune responses [24–29]. HSPA4 belongs to the HSP110 family and is related to tumor progression and outcomes [30–34]. Futhermore, the levels of multiple HSPs including HSPA4 were significantly enhanced in hepatocellular carcinoma (HCC), CRC and head and neck cancer compared with normal tissues. In vitro experiments showed that HSPA4 modulate the proliferation and migration of HCC and CRC cells, which presumably underpins the prognostic implication of HSPA4 in CRC [32]. In CRC cell lines, HSPA4 knockdown suppressed cell proliferation and migration, and caused arrest in the G2-phase of the cell cycle along with increased levels of apoptosis by inhibiting the activation of the PI3K/Akt signaling pathway and reducing the cell cycle progression markers CCND1 and CDK6 [32]. In our study, HSPA4 was a hub gene and highly expressed in the TR group compared to the PR group, which was in line with the results of the external mRNA datasets. Using GSCALite, we found that HSPA4 was capable of activating the cell cycle in RC, in accordance with a previous CRC cell experiment [32]. Therefore, we assumed that HSPA4 increased the treatment sensitivity by promoting cell-cycle transitions of tumor cells, resulting in superior response to nCRT in the TR group with higher HSPA4 levels.

NIPSNAP1 a kind of mitochondrial matrix protein, could recruit autophagy receptors and play a role in PARKIN-dependent mitophagy in damaged mitochondria [35]. NIPSNAP1 deficiency was found to be related to the accumulation of reactive oxygen species (ROS) and mitophagy deficiency in the brain of zebrafish larvae [36]. Since ROS generated in mitochondria was a strong inducer of apoptosis during chemo- or radiotherapy, mitophagy could degrade dysfunctional mitochondria to decrease ROS production, which promoted cell survival and resisted apoptosis during chemotherapy in various chemo-resistant cancer cells [37–41]. In CRC, it had been reported that mitophagy contributed to doxorubicin resistance in HCT8 cancer stem cells, and inhibition of mitophagy enhanced doxorubicin sensitivity [42]. Thus, reversing mitophagy-mediated protective mechanisms might be one of many ways to reverse chemotherapy resistance. In this study, the NIPSNAP1 level was observed to be higher in the PR than in the TR group. Due to limited literature reports, few interactions of NIPSNAP1 with other DEPs were found in our results. However, it could be speculated that, (1) since mitophagy had been linked to drug resistance in CRC and (2) NIPSNAP1 had been shown to affect mitophagy and ROS production in other types of cancer, that mitophagy impairment and elevated ROS production might exist in the TR group due to the lower level of NIPSNAP1, which partially explains their greater sensitivity to nCRT. SPTB is involved in erythrocyte membrane stability, and the mutations in this gene have been implicated in spherocytosis type 2 and hereditary elliptocytosis [43–45]. However, few studies have reported on the association of between SPTB and solid tumors.

To there, the exploration of molecular biomarkers in response to nCRT in RC mainly focused on proteomics, transcriptomics, DNA mutation and methylation, and single nucleotide polymorphisms (SNPs) [8]. We selected proteomics rather than transcriptomics to search biomarkers in the study, as the changes in protein could reflect the response to nCRT more directly compared with mRNA. Meta-analysis suggested that variation in mRNA levels is often a poor predictor of changes in protein abundance [46]. In addition, according to another proteomic study on CRC cell lines, proteomic data might provide better prediction of drug sensitivity in CRC when compared to genomic and transcriptomic profiles in informing personalized cancer treatment [47]. Although the mRNA level is not always consistent with protein abundance—probably due to the impact on the binding, processing or translation of mRNA, it was found that genes with stable mRNA and protein expression tend to have higher mRNA-protein correlation [48]. The three proteins in this study were identified and validated at both the protein and mRNA levels, indicating that they might be stable markers in patients with LARC. With respect to the interference of endogenous expression, consistent expression differences of the markers were found at protein and mRNA levels in the two distinct cohorts, and significant linear trends of identified markers were found across different treatment responses in the total internal cohort, suggesting the inference of endogenous expressions might be few. Additionally, we searched on public data resources and literature, and no report on endogenous expression of the three markers had been found in rectal cancer patients. Based on these findings, although we could not completely exclude the interference from endogenous expression, we speculated that endogenous expressions of the identified markers in specific patients occurred with a low probability. After validation in larger independent cohorts, protein markers could be applied to clinical use more promptly through mature protein detection technology (e.g., immunohistochemistry for biopsy samples) for the prediction of response to nCRT—which is more cost-effective and simpler to implement.

In addition to tissue-based proteomics, blood-based proteomics is also a common approach to research the response to nCRT in RC [8]. However, the results of blood-derived proteomics are not always satisfying. Proteins from tumor tissues are most likely subjected to interference when released into the blood, combined with multiple variables introduced by the MS procedure and sample processing—confounding differential proteins identification. We dissected tissue-based proteomics in search of protein markers to predict the response to nCRT in RC for two reasons: (1) tissue-based proteomics data directly reveal the differences in the tumor tissues themself; and (2) the differential proteins identified in tumor tissues could be verified using plasma samples from patients with LARC in a future study. Nevertheless, other similar studies had smaller sample sizes, inconsistent TRG grading, and different thresholds for screening differential proteins. Until now, few overlapping proteins have been found between studies, and most lack verification [49]. In the case of different evaluation systems and data types, we proved that specific proteins (HSPA4, NIPSNAP1, and SPTB) and their combinations had a favorable performance as predictive markers for nCRT response. The combination of the two markers, HSPA4 and SPTB, could achieve relatively higher AUCs in the internal discovery cohort (AUC = 0.980) and external validation cohort (AUC = 0.741), as well as in the total internal discovery cohort (TR versus MR & PR: AUC = 0.849, TR & MR versus PR: AUC = 0.836), with the TNR and TPR almost all above 0.7. Simultaneously, the combination of HSPA4 and SPTB also demonstrated good TPR (0.91) when comparing the good and moderate response group with the poor response group—potentially screening patients with poor benefit to nCRT to a greater extent and avoiding unnecessary pre-operative treatment. Next, for screening out patients who might achieve total response to nCRT more sensitively, the combination of NIPSNAP1 and SPTB could reach a TNR of 1.00 when comparing patients with total response to others. This might help more patients receive nCRT and get surgery opportunities with organ preservation. Additionally, the levels of the three proteins linearly varied with the degrees of therapy response. Thus, the three proteins, including their optional combinations, were considered viable biomarkers for the prediction of nCRT response and deserve further investigation for their predictive value. Furthermore, based on our findings, their underlying mechanisms and potential as targets for treatment sensitization should also be elucidated in future research.

This was a single-center retrospective study with limited cases. Due to the lack of definite diagnosis and unclear therapeutic efficacy, multiple patients were sufferering from LARC. Few rectal patients could receive an accurate diagnosis of LARC and received nCRT regardless. Therefore, it was crucial to shed light on the prediction of nCRT response for treatment decisions. Our research institution is part of a national hospital with patients from all over the country, which helped to offset the single population—although undeniably still a single center. As part of future studies, we continue to collect samples to validate biomarker feasibility in future multi-center studies with expanded sample sizes.

## Conclusions

The results of the present study illustrate the difference in tissue proteomics between patients with LARC based on their response to nCRT. These are preliminary results based on a small cohort, however, the results suggest that the tumor tissue-derived proteins (HSPA4, SPTB and NIPSNAP1) and a combination of HSPA4 and SPTB, could achieve relatively high predictive ability for nCRT sensitivity. Therefore, these proteins and their optional combinations might be potential biomarkers to predict nCRT response in patients with LARC. Furthermore, the combination of HSPA4 and SPTB also demonstrated good TPR (0.91) between the poor response group and the others, which could be useful to screen patients with a lower likelihood to benefit from nCRT and avoid unnecessary pre-operative treatment. Moreover, the combination of NIPSNAP1 and SPTB could reach a TNR of 1.00 for patients with total response, which might help identify patients likely to achieve total response to nCRT. Our study provides insight into potential biomarker identification and pathways to determine nCRT response sensitivity in a Chinese cohort of patients with LARC.

## Supplementary Information

**Additional file 1: Table S1.** List of differentially expressed proteins with higher expression in TR group compared with PR group

**Additional file 2: Table S2.** List of differentially expressed proteins with lower expression in TR group compared with PR group

## Data Availability

Qualified researchers may request access to raw data and materials by sending a request to the corresponding author. Data will be shared after approval of a proposal, with a signed data access agreement.

## Abbreviations

5-FU: 5-Fluorouracil
AJCC: American Joint Committee on Cancer
AUC: Area under the curve
AV: Anal verge
CRC: Colorectal cancer
CT: Computed tomography
CTRP: Cancer Therapeutics Response Portal
DEPs: Differentially expressed proteins
DDA: Data dependent acquisition
DTT: Dithiothreitol
EMT: Epithelial–mesenchymal transition
FASP: Filter-aided sample preparation
FFPE: Formalin-fixed paraffin-embedded
GDSC: Genomics of Drug Sensitivity in Cancer
GO: Gene ontology
GSEA: Gene set enrichment analysis
HCC: Hepatocellular carcinoma
HSPA4: Heat shock proteins family A member 4
HSPs: Heat shock proteins
IAA: Iodoacetamide
KEGG: Kyoto Encyclopedia of Genes and Genomes
LARC: Locally advanced rectal cancer
LC–MS/MS: Liquid chromatography with tandem mass spectrometry
LCRT: Long course radiotherapy
MR: Moderate responders
MRI: Magnetic resonance imaging
nCRT: Neoadjuvant chemoradiation therapy
NES: Normalized Enrichment Scores
NIPSNAP1: Nitrophenylphosphatase domain and non-neuronal SNAP25-like protein homolog 1
pCR: Pathologic complete response
PLS-DA: Partial least squares discriminant analysis
PPI: Protein–protein interaction
PR: Poor responders
RC: Rectal cancer
RECIST: Response Evaluation Criteria in Solid Tumors
ROC: Receiver operating characteristic
ROS: Reactive oxygen species
RPPA: Reverse phase protein array
SNP: Single nucleotide polymorphism
SPTB: Spectrin beta, erythrocytic
TCPA: The Cancer Proteome Atlas
TME: Total mesorectal excision
TNR: True negative rate
TPR: True positive rate
TR: Total responders
TRG: Tumor regression grade

## References

[1] Sung H, Ferlay J, Siegel RL, Laversanne M, Soerjomataram I, Jemal A (2021). Global Cancer Statistics 2020: GLOBOCAN estimates of incidence and mortality worldwide for 36 cancers in 185 countries. CA Cancer J Clin.

[2] Bosset JF, Collette L, Calais G, Mineur L, Maingon P, Radosevic-Jelic L (2006). Chemotherapy with preoperative radiotherapy in rectal cancer. N Engl J Med.

[3] Ortholan C, Romestaing P, Chapet O, Gerard JP (2012). Correlation in rectal cancer between clinical tumor response after neoadjuvant radiotherapy and sphincter or organ preservation: 10-year results of the Lyon R 96–02 randomized trial. Int J Radiat Oncol Biol Phys.

[4] Park IJ, You YN, Agarwal A, Skibber JM, Rodriguez-Bigas MA, Eng C (2012). Neoadjuvant treatment response as an early response indicator for patients with rectal cancer. J Clin Oncol.

[5] Braendengen M, Tveit KM, Berglund A, Birkemeyer E, Frykholm G, Påhlman L (2008). Randomized phase III study comparing preoperative radiotherapy with chemoradiotherapy in nonresectable rectal cancer. J Clin Oncol.

[6] Roh MS, Colangelo LH, O'Connell MJ, Yothers G, Deutsch M, Allegra CJ (2009). Preoperative multimodality therapy improves disease-free survival in patients with carcinoma of the rectum: NSABP R-03. J Clin Oncol.

[7] Smith KD, Tan D, Das P, Chang GJ, Kattepogu K, Feig BW (2010). Clinical significance of acellular mucin in rectal adenocarcinoma patients with a pathologic complete response to preoperative chemoradiation. Ann Surg.

[8] Dayde D, Tanaka I, Jain R, Tai MC, Taguchi A (2017). Predictive and prognostic molecular biomarkers for response to neoadjuvant chemoradiation in rectal cancer. Int J Mol Sci.

[9] Timmerman C, Taveras LR, Huerta S (2018). Clinical and molecular diagnosis of pathologic complete response in rectal cancer: an update. Expert Rev Mol Diagn.

[10] Qiu HZ, Wu B, Xiao Y, Lin GL (2011). Combination of differentiation and T stage can predict unresponsiveness to neoadjuvant therapy for rectal cancer. Colorectal Dis.

[11] Bitterman DS, Resende Salgado L, Moore HG, Sanfilippo NJ, Gu P, Hatzaras I (2015). Predictors of complete response and disease recurrence following chemoradiation for rectal cancer. Front Oncol.

[12] Lahaye MJ, Engelen SM, Nelemans PJ, Beets GL, van de Velde CJ, van Engelshoven JM (2005). Imaging for predicting the risk factors–the circumferential resection margin and nodal disease–of local recurrence in rectal cancer: a meta-analysis. Semin Ultrasound CT MR.

[13] Memon S, Lynch AC, Bressel M, Wise AG, Heriot AG (2015). Systematic review and meta-analysis of the accuracy of MRI and endorectal ultrasound in the restaging and response assessment of rectal cancer following neoadjuvant therapy. Colorectal Dis.

[14] Trakarnsanga A, Gönen M, Shia J, Nash GM, Temple LK, Guillem JG (2014). Comparison of tumor regression grade systems for locally advanced rectal cancer after multimodality treatment. J Natl Cancer Instit..

[15] Fekete JT, Győrffy B (2019). ROCplot.org: validating predictive biomarkers of chemotherapy/hormonal therapy/anti-HER2 therapy using transcriptomic data of 3,104 breast cancer patients. Int J Cancer.

[16] Liu C, Hu F, Xia M, Han L, Zhang Q, Guo A (2018). GSCALite: a web server for gene set cancer analysis. Bioinformatics.

[17] Shannon P, Markiel A, Ozier O, Baliga NS, Wang JT, Ramage D (2003). Cytoscape: a software environment for integrated models of biomolecular interaction networks. Genome Res.

[18] Nie K, Shi L, Chen Q, Hu X, Jabbour SK, Yue N (2016). Rectal cancer: assessment of neoadjuvant chemoradiation outcome based on radiomics of multiparametric MRI. Clin Cancer Res.

[19] Del Puerto-Nevado L, Marin-Arango JP, Fernandez-Aceñero MJ, Arroyo-Manzano D, Martinez-Useros J, Borrero-Palacios A (2016). Predictive value of vrk 1 and 2 for rectal adenocarcinoma response to neoadjuvant chemoradiation therapy: a retrospective observational cohort study. BMC Cancer.

[20] Wang Y, Yang L, Bao H, Fan X, Xia F, Wan J (2021). Utility of ctDNA in predicting response to neoadjuvant chemoradiotherapy and prognosis assessment in locally advanced rectal cancer: a prospective cohort study. PLoS Med.

[21] Xiao WW, Li M, Guo ZW, Zhang R, Xi SY, Zhang XG (2021). A genotype signature for predicting pathologic complete response in locally advanced rectal cancer. Int J Radiat Oncol Biol Phys.

[22] Liu YP, Zheng CC, Huang YN, He ML, Xu WW, Li B (2021). Molecular mechanisms of chemo- and radiotherapy resistance and the potential implications for cancer treatment. MedComm.

[23] Ozdian T, Holub D, Maceckova Z, Varanasi L, Rylova G, Rehulka J (2017). Proteomic profiling reveals DNA damage, nucleolar and ribosomal stress are the main responses to oxaliplatin treatment in cancer cells. J Proteomics.

[24] Ciocca DR, Calderwood SK (2005). Heat shock proteins in cancer: diagnostic, prognostic, predictive, and treatment implications. Cell Stress Chaperones.

[25] Kennedy D, Jäger R, Mosser DD, Samali A (2014). Regulation of apoptosis by heat shock proteins. IUBMB Life.

[26] Calderwood SK, Gong J (2016). Heat shock proteins promote cancer: it's a protection racket. Trends Biochem Sci.

[27] Calderwood SK, Khaleque MA, Sawyer DB, Ciocca DR (2006). Heat shock proteins in cancer: chaperones of tumorigenesis. Trends Biochem Sci.

[28] Zhang Z, Jing J, Ye Y, Chen Z, Jing Y, Li S (2020). Characterization of the dual functional effects of heat shock proteins (HSPs) in cancer hallmarks to aid development of HSP inhibitors. Genome Med.

[29] Srivastava P (2002). Interaction of heat shock proteins with peptides and antigen presenting cells: chaperoning of the innate and adaptive immune responses. Annu Rev Immunol.

[30] Yang Z, Zhuang L, Szatmary P, Wen L, Sun H, Lu Y (2015). Upregulation of heat shock proteins (HSPA12A, HSP90B1, HSPA4, HSPA5 and HSPA6) in tumour tissues is associated with poor outcomes from HBV-related early-stage hepatocellular carcinoma. Int J Med Sci.

[31] Gu Y, Liu Y, Fu L, Zhai L, Zhu J, Han Y (2019). Tumor-educated B cells selectively promote breast cancer lymph node metastasis by HSPA4-targeting IgG. Nat Med.

[32] Zhang M, Dai W, Li Z, Tang L, Chen J, Chen C (2021). HSPA4 knockdown retarded progression and development of colorectal cancer. Cancer Manage Res.

[33] Fan G, Tu Y, Wu N, Xiao H (2020). The expression profiles and prognostic values of HSPs family members in Head and neck cancer. Cancer Cell Int.

[34] Wu CY, Lin CT, Wu MZ, Wu KJ (2011). Induction of HSPA4 and HSPA14 by NBS1 overexpression contributes to NBS1-induced in vitro metastatic and transformation activity. J Biomed Sci.

[35] Princely Abudu Y, Pankiv S, Mathai BJ, Håkon Lystad A, Bindesbøll C, Brenne HB (2019). NIPSNAP1 and NIPSNAP2 act as "Eat Me" signals for mitophagy. Dev Cell.

[36] Abudu YP, Pankiv S, Mathai BJ, Lamark T, Johansen T, Simonsen A (2019). NIPSNAP1 and NIPSNAP2 act as "eat me" signals to allow sustained recruitment of autophagy receptors during mitophagy. Autophagy.

[37] Panigrahi DP, Praharaj PP, Bhol CS, Mahapatra KK, Patra S, Behera BP (2020). The emerging, multifaceted role of mitophagy in cancer and cancer therapeutics. Semin Cancer Biol.

[38] Ianniciello A, Rattigan KM, Helgason GV (2018). The Ins and Outs of autophagy and metabolism in hematopoietic and leukemic stem cells: food for thought. Front Cell Dev Biol.

[39] Levy A, Stedman A, Deutsch E, Donnadieu F, Virgin HW, Sansonetti PJ (2020). Innate immune receptor NOD2 mediates LGR5(+) intestinal stem cell protection against ROS cytotoxicity via mitophagy stimulation. Proc Natl Acad Sci USA.

[40] Xie Y, Liu J, Kang R, Tang D (2020). Mitophagy receptors in tumor biology. Front Cell Dev Biol.

[41] Villa E, Proïcs E, Rubio-Patiño C, Obba S, Zunino B, Bossowski JP (2017). Parkin-independent mitophagy controls chemotherapeutic response in cancer cells. Cell Rep.

[42] Yan C, Luo L, Guo CY, Goto S, Urata Y, Shao JH (2017). Doxorubicin-induced mitophagy contributes to drug resistance in cancer stem cells from HCT8 human colorectal cancer cells. Cancer Lett.

[43] Tole S, Dhir P, Pugi J, Drury LJ, Butchart S, Fantauzzi M (2020). Genotype-phenotype correlation in children with hereditary spherocytosis. Br J Haematol.

[44] Park J, Jeong DC, Yoo J, Jang W, Chae H, Kim J (2016). Mutational characteristics of ANK1 and SPTB genes in hereditary spherocytosis. Clin Genet.

[45] Maillet P, Alloisio N, Morlé L, Delaunay J (1996). Spectrin mutations in hereditary elliptocytosis and hereditary spherocytosis. Hum Mutat.

[46] Jarnuczak AF, Najgebauer H, Barzine M, Kundu DJ, Ghavidel F, Perez-Riverol Y (2021). An integrated landscape of protein expression in human cancer. Scientific data.

[47] Wang J, Mouradov D, Wang X, Jorissen RN, Chambers MC, Zimmerman LJ (2017). Colorectal cancer cell line proteomes are representative of primary tumors and predict drug sensitivity. Gastroenterology.

[48] Zhang B, Wang J, Wang X, Zhu J, Liu Q, Shi Z (2014). Proteogenomic characterization of human colon and rectal cancer. Nature.

[49] Alves Martins BA, de Bulhões GF, Cavalcanti IN, Martins MM, de Oliveira PG, Martins AMA (2019). Biomarkers in colorectal cancer: the role of translational proteomics research. Front Oncol.

