# Supplementary material for: Identification of proteomic markers for prediction of the response to 5-Fluorouracil based neoadjuvant chemoradiotherapy in locally advanced rectal cancer patients
Source: Cancer Cell Int. 2022 Mar 15;22:117. doi: 10.1186/s12935-022-02530-0 (PMC8922748; doi:10.1186/s12935-022-02530-0)
Supplement: Supplementary file 1 — Additional file 1: Table S1. List of differentially expressed proteins with higher expression in TR group compared with PR group [file 12935_2022_2530_MOESM1_ESM.docx]

**Table S1: List of differentially expressed proteins with higher expression in TR group compared with PR group**

| **UniprotKB ID** | ***p*-value** | **Log2FoldChange**  **(TR/PR)** | **UniprotKB ID** | ***p*-value** | **Log2FoldChange**  **(TR/PR)** |
| --- | --- | --- | --- | --- | --- |
| Q9Y3B4 | 0.007 | 2.195 | Q9Y2X3 | 0.043 | 0.944 |
| P00167 | 0.043 | 1.857 | O14980 | 0.015 | 0.942 |
| Q06136 | 0.005 | 1.636 | E9PHI4 | 0.034 | 0.938 |
| Q99436 | 0.039 | 1.462 | P49790 | 0.018 | 0.937 |
| A0A494C0R8 | 0.027 | 1.441 | P18077 | 0.044 | 0.930 |
| Q96P70 | 0.015 | 1.407 | P22087 | 0.014 | 0.897 |
| Q9NWB6 | 0.050 | 1.324 | O60506 | 0.010 | 0.896 |
| F8W727 | 0.013 | 1.309 | Q96ST3 | 0.017 | 0.869 |
| Q99615 | 0.008 | 1.284 | P43490 | 0.035 | 0.812 |
| Q92769 | 0.018 | 1.210 | Q9NR30 | 0.023 | 0.807 |
| Q93050 | 0.050 | 1.176 | P11940 | 0.041 | 0.807 |
| Q86YV0 | 0.022 | 1.165 | P61964 | 0.039 | 0.777 |
| P62495 | 0.014 | 1.165 | H7C2Q8 | 0.011 | 0.762 |
| P42285 | 0.025 | 1.163 | P49915 | 0.023 | 0.761 |
| Q96S66 | 0.031 | 1.125 | P34932 | 0.011 | 0.750 |
| Q14738 | 0.022 | 1.107 | A0A0A6YYL6 | 0.002 | 0.749 |
| O60504 | 0.047 | 1.087 | P46063 | 0.001 | 0.731 |
| P61086 | 0.011 | 1.079 | Q9Y230 | 0.011 | 0.702 |
| Q9NWV4 | 0.044 | 1.078 | P29350 | 0.035 | 0.684 |
| O75844 | 0.012 | 1.076 | Q7L1Q6 | 0.049 | 0.683 |
| Q15043 | 0.014 | 1.072 | Q9NZM1 | 0.023 | 0.642 |
| Q92696 | 0.020 | 1.063 | Q14697 | 0.043 | 0.635 |
| Q15393 | 0.025 | 0.983 | Q9Y3I0 | 0.015 | 0.626 |

TR, total responders; PR, poor responders.
