# Supplementary material for: Identification of proteomic markers for prediction of the response to 5-Fluorouracil based neoadjuvant chemoradiotherapy in locally advanced rectal cancer patients
Source: Cancer Cell Int. 2022 Mar 15;22:117. doi: 10.1186/s12935-022-02530-0 (PMC8922748; doi:10.1186/s12935-022-02530-0)
Supplement: Supplementary file 2 — Additional file 2: Table S2. List of differentially expressed proteins with lower expression in TR group compared with PR group [file 12935_2022_2530_MOESM2_ESM.docx]

**Table S2: List of differentially expressed proteins with lower expression in TR group compared with PR group**

| **UniprotKB ID** | ***p*-value** | **Log2FoldChange**  **(TR/PR)** | **UniprotKB ID** | ***p*-value** | **Log2FoldChange**  **(TR/PR)** |
| --- | --- | --- | --- | --- | --- |
| Q6PCB0 | 0.024 | -2.279 | P55263 | 0.010 | -1.174 |
| Q16775 | 0.012 | -2.192 | Q9Y6R7 | 0.020 | -1.154 |
| P02730 | 0.002 | -2.029 | P55899 | 0.022 | -1.142 |
| P16157 | 0.004 | -1.996 | P68871 | 0.012 | -1.115 |
| P11277 | 0.010 | -1.778 | P14543 | 0.019 | -1.023 |
| Q9BPW8 | 0.011 | -1.745 | A0A2R8YFX0 | 0.013 | -0.986 |
| P69905 | 0.000 | -1.738 | P21397 | 0.047 | -0.978 |
| O00292 | 0.005 | -1.701 | Q9H008 | 0.021 | -0.976 |
| Q9Y3B8 | 0.034 | -1.695 | P11047 | 0.034 | -0.970 |
| P05165 | 0.015 | -1.668 | Q08043 | 0.028 | -0.961 |
| Q8TDX7 | 0.047 | -1.487 | H7BYY1 | 0.048 | -0.898 |
| P00915 | 0.015 | -1.468 | Q9Y678 | 0.003 | -0.891 |
| A0A2R8Y7X9 | 0.038 | -1.389 | Q9H7Z7 | 0.038 | -0.868 |
| P02042 | 0.004 | -1.387 | J3KN66 | 0.012 | -0.810 |
| Q9GZS9 | 0.039 | -1.373 | Q92820 | 0.029 | -0.793 |
| P07148 | 0.007 | -1.348 | A0A087WZ40 | 0.037 | -0.777 |
| A0A1B0GUN5 | 0.007 | -1.310 | Q8NFV4 | 0.023 | -0.777 |
| P40306 | 0.026 | -1.291 | Q9H444 | 0.030 | -0.773 |
| P02461 | 0.031 | -1.241 | Q9P2X0 | 0.005 | -0.738 |
| P10253 | 0.039 | -1.235 | H0YHG0 | 0.049 | -0.735 |
| Q15063 | 0.023 | -1.232 | P62316 | 0.043 | -0.626 |
| Q0VD83 | 0.008 | -1.198 | P06703 | 0.037 | -0.621 |
| O43488 | 0.028 | -1.183 |  |  |  |

TR, total responders; PR, poor responders.
